# Tomato Seed Coat Permeability to Selected Carbon Nanomaterials and Enhancement of Germination and Seedling Growth

**DOI:** 10.1155/2015/419215

**Published:** 2015-10-01

**Authors:** Tatsiana A. Ratnikova, Ramakrishna Podila, Apparao M. Rao, Alan G. Taylor

**Affiliations:** ^1^School of Integrative Plant Science, New York State Agricultural Experiment Station, Cornell University, Geneva, NY 14456-0462, USA; ^2^Department of Physics and Astronomy, Clemson University, Clemson, SC 29634, USA; ^3^Clemson Nanomaterials Center and COMSET, Clemson University, Clemson, SC 29634, USA

## Abstract

Seed coat permeability was examined using a model that tested the effects of soaking tomato (*Solanum lycopersicon*) seeds in combination with carbon-based nanomaterials (CBNMs) and ultrasonic irradiation (US). Penetration of seed coats to the embryo by CBNMs, as well as CBNMs effects on seed germination and seedling growth, was examined. Two CBNMs, C_60_(OH)_20_ (fullerol) and multiwalled nanotubes (MWNTs), were applied at 50 mg/L, and treatment exposure ranged from 0 to 60 minutes. Bright field, fluorescence, and electron microscopy and micro-Raman spectroscopy provided corroborating evidence that neither CBNM was able to penetrate the seed coat. The restriction of nanomaterial (NM) uptake was attributed to the semipermeable layer located at the innermost layer of the seed coat adjacent to the endosperm. Seed treatments using US at 30 or 60 minutes in the presence of MWNTs physically disrupted the seed coat; however, the integrity of the semipermeable layer was not impaired. The germination percentage and seedling length and weight were enhanced in the presence of MWNTs but were not altered by C_60_(OH)_20_. The combined exposure of seeds to NMs and US provided insight into the nanoparticle-seed interaction and may serve as a delivery system for enhancing seed germination and early seedling growth.

## 1. Introduction

Nanotechnology has undergone significant developments in recent years due to the potential applications of NMs in several fields such as electronics, therapeutics, imaging, sensing, environmental remediation, and consumer products [1]. CBNMs are one of the most established and most widely used NMs [1], and this rapid development with great economic potential of CBNMs has resulted in research on biological and environmental systems and in particular on human health effects. Our discussion will focus on plants and in particular the effects of CBNMs on seed coat permeability, germination, and early seedling development.

Positive, neutral, and adverse effects were reported for a range of plant species exposed to NMs at different developmental stages [2–17]. Of these plant science studies, few were conducted on the effect of NMs on seed germination [4–8]. As this finding may have significant improvement in crop productivity, research is needed on the applications of nanotechnology to seeds. However, it remains unclear how NMs can affect seed germination and seedling growth. CBNTs were stated to penetrate tomato seed coats and enhanced seed germination and seedling growth by increasing imbibition rates [5] and activation of water channel proteins [9]. Also, CBNMs increased the water and essential calcium and iron nutrient uptake efficiency that may enhance germination and plant growth and development in tomato plants [9]. In addition, MWNTs were shown to improve not only water uptake capacity and biomass, but also flowering and fruit yield [10]. However, the penetration of CBNMs into seeds could be restricted due to the morphology and composition of the seed coat enclosing the embryo [18]. In support of this, the tomato seeds possess a waxy, semipermeable layer as the innermost part of their seed coat [19]. This layer was shown to be permeable only to nonionic, moderately lipophilic compounds, while acting as a barrier to water-soluble compounds [20, 21]. Therefore, whether NMs can penetrate seed coats needs clarification.

One method to treat seeds is by soaking in aqueous solutions for short periods of time to allow imbibition [22]. CBNMs could be applied in a seed soak but should be kept suspended during application. Two major CBNMs, hydrophilic fullerols and hydrophobic MWNTs, were used to treat seeds due to their contrasting physiochemical properties, and ultrasound was used to suspend MWNTs in aqueous preparations. In addition, treatment of seeds by US only was reported to improve germination rate and seedling growth [23–27]. With respect to tomato, US was reported to enhance seed germination [27]. In our study, a double exposure seed treatment model with US with CBNMs was examined to investigate the ability of CBNMs to penetrate a tomato seed coat. The rationale of this model was an attempt to overcome the physical barrier of tomato seed coat by CBNMs during US.

## 2. Materials and Methods

### 2.1. Seed Treatment Protocol

Tomato seeds (*Solanum lycopersicon*, variety Talladega) were used in all experiments. The seeds were from Syngenta Seeds, Inc., and seeds were maintained at 4°C until being required. Prior to experimentation, the seeds were surface sterilized via a 10 min treatment of 2.5% sodium hypochlorite and then rinsed five times with MilliQ water (MilliQ).

The double exposure effect of US and CBNMs (US-CBNMs double exposure) on tomato seeds was investigated according to the following procedures. A total of 20 treatments were performed on tomato seeds: MilliQ, gallic acid (GA), C_60_(OH)_20_, MWNT-GA, and seed treatments with each applied at five sonication times of 0, 5, 10, 30, or 60 min. Bright field microscopy images of the 20 seed treatments were conducted with an Imager A1, Zeiss microscope. The total soak time for all samples was 60 min. For example, for sample C_60_(OH)_20_ 5, seeds were sonicated for 5 minutes with C_60_(OH)_20_, followed by 55 min of seed soak in the same suspension without sonication. The relative controls (MilliQ 0, GA 0, C_60_(OH)_20_ 0, and MWNT-GA 0) were the seeds soaked in the suspensions for 60 min and not sonicated. The concentration of 50 mg/L of CBNMs and GA was used in all experiments, due to the previously reported positive effects of this NM concentration on plant development, good stability of suspensions, and smaller chance of nanoparticle aggregation and precipitation [2].

The hydrophobic nature of MWNTs required the presence of an amphiphylic-solubilizing factor for a stable suspension. GA, a type of phenolic acid, was chosen to solubilize NMs and enhance their bioavailability. The description and characterization of C_60_(OH)_20_, MWNTs, and GA, used as the dispersion agent for MWNTs, is presented in the Supplemental Information available online at http://dx.doi.org/10.1155/2015/419215 (Figure S1, Table S1).

Ultrasonication was conducted using a VC 130 PB US generator set at 20 kHz (Sonic & Materials, Inc.). All experiments were performed in 12 mL glass tubes (Fisher Scientific, Cat. # 14-957-76F), containing 150 tomato seeds dispersed in 6 mL of solution, with direct sonication (probe system). The sonication was performed in the continuous mode at 8 W. The tip of the horn was immersed into approximately 2 cm of solution, and samples were processed at a constant temperature of 24 ± 3°C. The temperature of water circulating in a water bath was set and the temperature within the glass tube was checked before and after sonication, so that the temperature of the samples remained constant during sonication.

### 2.2. Seed Coat Permeability Test, SEM, and Micro-Raman Spectroscopy

Thirty seeds for each treatment were placed on the top of agarose gel amended with Rhodamine B (Rhod B) (Sigma-Aldrich, lot 252425) termed Rhod B agar for approximately 9-10 hours at 22°C as described by our group earlier [20, 21]. After tomato seeds were cut longitudinally along the flat plane of each seed, embryos were manually extracted and examined by fluorescence microscopy (Imager A1, Zeiss). Specifically, to prepare Rhod B agar, 50 mg of Rhod B was dissolved into 100 mL of dH_2_O and mixed with 150 mL of melted 1.0% w/v agarose. 25 mL of Rhod B agar suspension was poured into 9 cm glass Petri dish and cooled. The experiment was replicated twice resulting in a total of 60 seeds per treatment.

Ten seeds from each treatment were cut along the longitudinal axis and then prepared for the SEM by freeze-drying with dry ice for 24 h. To ensure an optimum dispersion of MWNT by GA, a drop (10 *μ*L) of the stock solution of MWNT-GA was placed directly on the stubs and allowed to dry overnight. SEM imaging was performed using a field emission SEM (FESEM), Hitachi 4800, microscope operating at 5 kV. Seed samples were evenly coated with a thin film of platinum (~5 nm) using a Hummer 6.2 sputtering system SEM of nanoparticle.

Detailed Raman scattering measurements were performed on CBNM treated seeds to confirm the presence of MWNTs. The samples were prepared similar to the SEM studies without the platinum coating. Raman mapping was performed using Ar^+^ ion excitation at 514.5 nm coupled to a Dilor XY triple grating spectrometer. A 50x objective was used to map the MWNT Raman signal in 1.5 *μ*m steps.

### 2.3. Germination, Imbibition, and Seed Weight Loss Tests

Germination experiments were conducted at 22°C in 9 cm glass Petri dishes (60 seeds per dish). All treated seeds were washed 3 times with MilliQ water in order to remove the majority of NMs from the seed surfaces. Seeds were placed on the top of 1% w/v agarose gel (Fine Chemicals & Reagents, Lot DS22707T). A treated seed was considered germinated when the root (radicle) was visible. MilliQ water was added to the Petri dish as needed. Germination counts were made at 2, 3, 4, and 5 days, and the percentage was calculated. The experiment was replicated seven times resulting in a total of 170 seeds per treatment.

A 0.50 g seed sample (*W* (initial)) (approximately 150 seeds) was treated as discussed previously in the Seed Treatment Protocol. After treatments, seeds were surface dried on paper towels for 5 min and reweighed (*W* (after 60-minute treatment)). Hereafter seeds were allowed to remain undisturbed on paper towel at room temperature for 48 hours, and the seed weight was measured (*W* (48 h after drying)). The percentage increase in seed wet weight due to imbibition and percentage weight loss due to removal of the seed coat by treatment after drying were calculated as follows:

(1) $$\begin{array}{c} \text{Percentage}\;\text{Increase}\;\left(\%\right)=\frac{W\;\left(\text{after}\;60\;\min\;\text{treatment}\right)-W\;\left(\text{initial}\right)}{W\;\left(\text{initial}\right)}\times 100\%, \end{array}$$

(2) $$\begin{array}{c} \text{Percentage}\;\text{Loss}\;\left(\%\right)=\frac{W\;\left(\text{initial}\right)-W\;\left(48\;\text{h}\;\text{after}\;\text{drying}\right)}{W\;\left(\text{initial}\right)}\times 100\%. \end{array}$$

### 2.4. Seedling Growth Tests

Thirty seeds per replicate were treated and all seed treatments were placed on the top of 1% w/v agarose gel for 2 weeks. The lengths of roots and shoots of 30 tomato seedlings were measured with a vernier caliper after 14 days. This experiment was replicated 10 times. After 14 days, the plants were removed from the agarose and dried at room temperature for 48 hours and the mass of a drop (10 *μ*L) of the stock solution of MWNT-GA was placed directly on the stubs and allowed to dry overnight. SEM imaging of seeds was performed using a field emission SEM (FESEM), Hitachi 4800, microscope operating at 5 kV. Seed samples were evenly coated with a thin film of platinum (~5 nm) using a Hummer 6.2 sputtering system SEM of nanoparticle.

### 2.5. Statistical Analyses

Analysis of variance (AOV) was performed on water uptake and loss, root and shoot length, and seedling weight of seedlings that had been subjected to four different treatments (MilliQ, GA, fullerol, and MWNT-GA) and five different sonication times (0 min, 5 min, 10 min, 30 min, and 60 min). The experiments were analyzed as a randomized complete block design with a 4 × 5 factorial arrangement of treatments, and mean separations were performed by LSD test at (*p* < 0.05). The AOV was performed with JMP version 9 software. Means and standard errors were calculated for the germination and seed coat permeability tests.

## 3. Results 

### 3.1. US-CBNMs Double Exposure Effect on Seed Morphology

**Figure 1:**
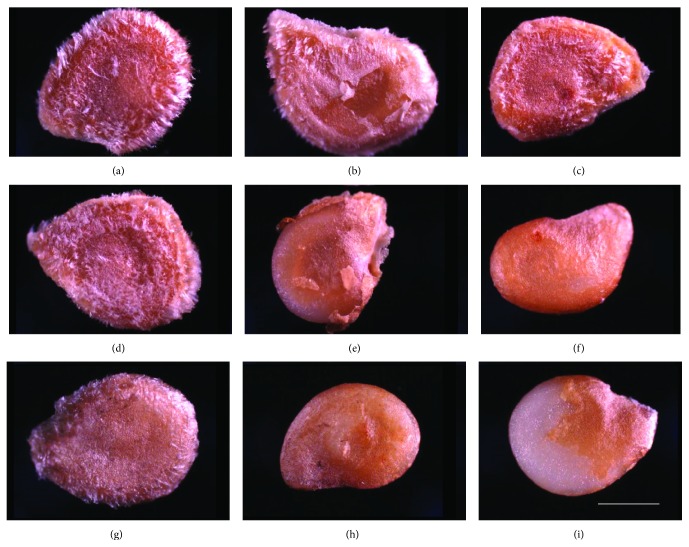
Bright field microscopy images of treated seeds. (a) MilliQ 0, (b) MilliQ 30, (c) MilliQ 60, (d) fullerol 0, (e) fullerol 30, (f) fullerol 60, (g) MWNT-GA 0, (h) MWNT-GA 30, and (i) MWNT-GA 60. Magnification: 2.5x. Scale bars for all of the images: 1 mm.

The effect of US time of 0, 30, and 60 min in combination with specific CBNMs treatments is shown in Figure 1. Some visible differences were observed for seeds exposed to MilliQ water (Figures 1(a)–1(c)) or GA (not shown). In comparison, the disruption of the outer seed coats was significant in the presence of C_60_(OH)_20_ (Figures 1(e) and 1(f)) and even more severe in the presence of MWNT-GA (Figures 1(h) and 1(i)). Noticeably, the US-CBNMs double exposure on seeds for extended periods led to the disruption and removal of seed coat tissue. An extreme example is presented in Figure 1(i), where the double exposure of the seeds to US and MWNT-GA for 60 min led to the removal of the entire outer seed coat, exposing the endosperm (Figure 1(i)).

**Figure 2:**
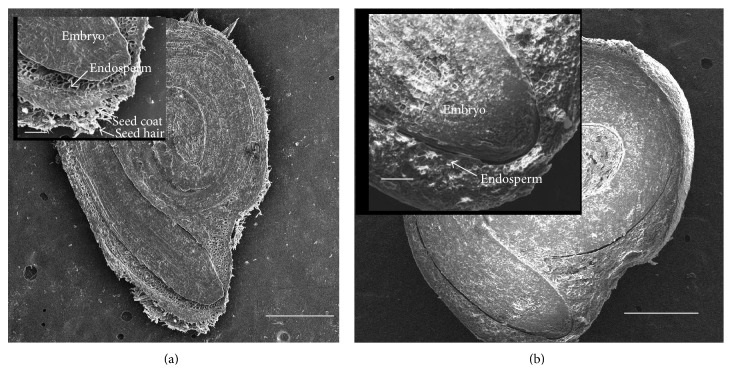
SEM images of cross section of tomato seed. (a) MilliQ 0 and (b) MWNT-GA 60. Insets: the radicle emergence region. Scale bars for all images were 500 *µ*m. Inset scale bar: 100 *µ*m.

The alteration of seed coat integrity was affected by the combination of CBNMs and US treatments (Figure 1). There were 12% small cracks in the seed coats after MilliQ 0, GA 0, C_60_(OH)_20_ 0, and MWNT-GA 0 treatments. In contrast, 60 min of US treatment resulted in 48, 56, 72, and 68% damaged seed coats after MilliQ 60, GA 60, C_60_(OH)_20_ 60, and MWNT-GA 60, respectively. To further illustrate the double exposure effects of US and MWNT-GA on seed coats, we conducted an SEM examination of seed cross sections (Figures 2(a) and 2(b)). The US-CBNM double exposure treatment removed seed coat hairs and eroded the seed coat.

### 3.2. US-CBNMs Double Exposure Effect on Seed Coat Permeability

**Figure 3:**
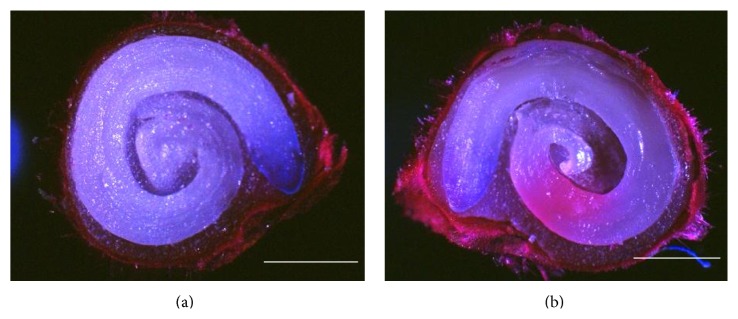
Cross section (overlay of bright field and fluorescence microscopy) of tomato seed. (a) MWNT-GA 60 tomato seed. (b) Previously punctured MWNT-GA 60 tomato seed. Scale bars: 500 *µ*m.

**Figure 4:**
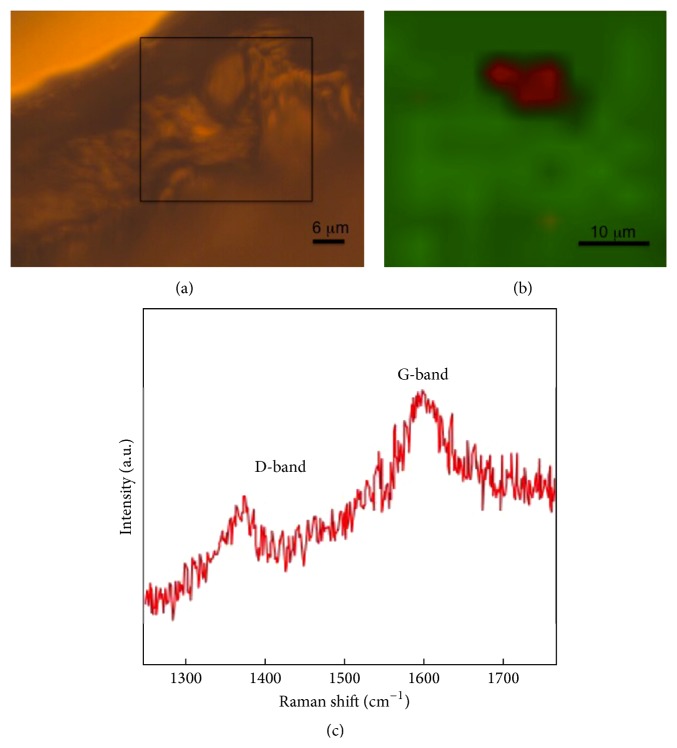
(a) A representative optical (bright field microscopy) image of the punctured seed cross section. Raman mapping was performed on the boxed area. (b) The Raman map of G-band intensity in the boxed region showing the presence of MWNTs. The red region represents a portion of the seed that exhibited the G-band signifying the presence of MWNTs, while the green region is devoid of MWNTs. (c) A typical Raman spectrum of MWNT includes a disorder band (or D-band) at 1350 cm^−1^ and a graphitic band (G-band) at 1585 cm^−1^.

In order to examine the effect of US and NMs on seed coat permeability, a detailed fluorescence microscopy study of cross sections from intact and punctured seeds was conducted. Punctured seeds were prepared by piercing the seed coat with a small gauge needle. The fluorescent Rhod B was not observed to diffuse from the agarose media to the embryo from the MWNT-GA 60 treatment (Figure 3(a)). However, Rhod B fluorescence was observed by first puncturing the tomato seed coat prior to MWNT-GA 60 (Figure 3(b)). Additionally, black aggregates of NMs were frequently found in the embryos of punctured seeds, indicating the transport of MWNT-GA into the embryo, while no evidence of NMs was observed from nonpunctured seeds (not shown). To quantify this phenomenon, 60 seeds for each treatment were examined under fluorescence microscopy (Table 1). The percentage of the intact treated seeds with Rhod B permeable seed coat was three percent or less, while 100 percent of the punctured treated seeds had permeable seed coats. To verify this observation, we performed Raman mapping measurements on the longitudinal sections of seed to confirm the presence of MWNTs. First, seeds incubated in MWNT suspension with nonpunctured seed coats did not show any Raman signal suggesting that MWNTs did not penetrate through the seed coat (data not shown). A representative Raman mapping image of punctured seed exposed to MWNTs is shown in Figures 4(a) and 4(b). MWNTs primarily exhibit a disorder band (or D-band) at 1350 cm^−1^ and a graphitic band (G-band) at 1585 cm^−1^ in their Raman spectrum (Figure 4(c)). Therefore, only punctured seeds showed the presence of MWNTs in the embryo.

### 3.3. US-CBNMs Double Exposure Effect on Seed Germination, Imbibition, and Seed Weight Loss

The effect of double exposure of US and CBNM seed treatments on tomato seed germination recorded after 5 days is presented in Table 2. A high quality seed lot was used in this study with 93% germination for the control, MilliQ 0 treatment; therefore, only small improvements in germination were possible. A short 5-minute US treatment for all treatments (MilliQ 5, GA 5, C_60_(OH)_20_ 5, and MWNT-GA 5) had higher percentage germination compared to the control—MilliQ 0. However, MilliQ-US treatment in excess of 5 minutes had a similar germination percentage to the MilliQ 0, thus demonstrating that US treatments were not detrimental to germination. Conversely, all samples exposed to MWNT-GA showed the maximum germination percentage of 99%, regardless of US treatment, suggesting a protective mechanism of MWNT-GA against prolonged exposure of seeds to US. No significant differences were measured in germination between treatments at days 2 and 3, and over 50 percent of the seeds had germinated by day 3 (data not shown), which indicated that the germination rate was not affected by CNNM seed treatment nor exposure duration.

**Figure 5:**
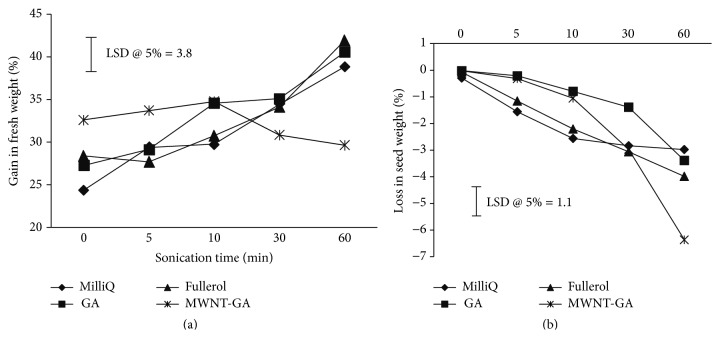
(a) The gain in seed wet weight after 0, 5, 10, 30, or 60 min US treatments in MilliQ, GA, C_60_(OH)_20_, and MWNT-GA. (b) The loss in dry seed weight 48 hours after drying from US treatment in MilliQ, GA, C_60_(OH)_20_, and MWNT-GA. Means separated by LSD bar are significantly different at *p* < 0.05. The total soak time for all treatments was 60 min. That is, 0 min of sonication is equivalent to 0 min of sonication plus 60 min of soak, 5 min sonication is equivalent to 5 min of sonication plus 55 min of soak in the solution, and so forth.

In order to better understand the effect of NMs on tomato seed germination, we further examined the percentage increase in seed weight immediately upon treatment (the percent increase of the seed wet weight was calculated according to formula (1)). As shown in Figure 5(a), 60 min of soaking in MilliQ, GA, C_60_(OH)_20_, and MWNT-GA without US resulted in a 24 (MilliQ 0), 27 (GA 0), 28 (C_60_(OH)_20_ 0), and 33% (MWNT-GA 0) increase in seed wet weight, respectively. There was a significant interaction between seed treatments and duration, and the seed weight increased to approximately 40% for the MilliQ 60, GA 60, and C_60_(OH)_20_ 60 treatments, while the MWNT-GA 60 followed a different pattern (Figure 5(a)). Specifically, the weight of MWNT-GA 60 seeds increased after short US exposures of 5 and 10 min to about 35%, followed by a decrease to 30% after 30 and 60 min. This biological phenomenon was attributed to the mechanical disruption of the seed coats produced by the interaction of sonication with MWNT-GA.

We further recorded the percent decrease or loss in the seed dry weight after drying for 48 hours (Figure 5(b)) (the percent decrease in the dry weight of the seeds was calculated according to formula (2)). A 60 min water soak only in all NM treatments did not decrease seed weight (percent decrease approximately 0%). There was a significant interaction between seed treatments and duration, and the 60 min of sonication treatments, MilliQ 60, GA 60, C_60_(OH)_20_ 60, and MWNT-GA 60, resulted in a 3, 3, 4, and 7% weight loss, respectively. Therefore, the MWNT-GA in combination with 60 min US treatment resulted in the greatest dry seed weight loss.

### 3.4. US-CBNMs Double Exposure Effect on Seedling Development

**Figure 6:**
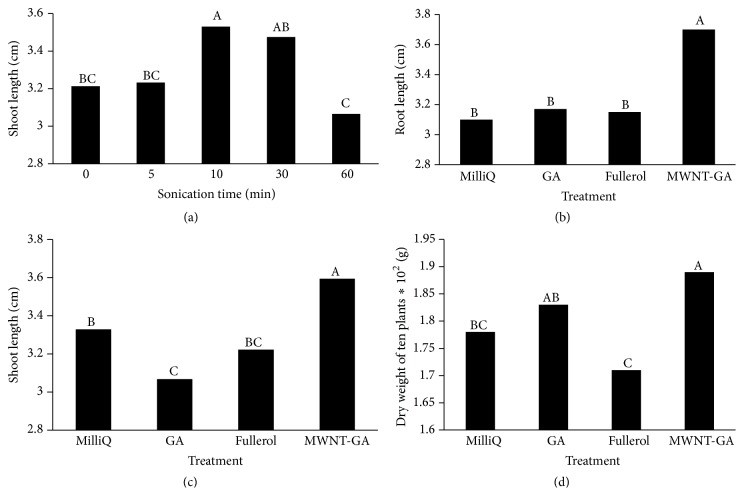
(a) Main effect of seedling shoot length versus US time. Main effects of root length (b), shoot length (c), and (d) dry weight of 10 plants versus seed treatment. All measurements made after 14 days. Means with the same letter are not significantly different by LSD (*p* < 0.05).

Application of MWNT-GA increased the root and shoot lengths and weight of tomato seedlings. The statistical analysis of this factorial arrangement of treatments revealed only main effects were significant, but not interactions for any seedling growth parameter. US duration had a significant effect on shoot length, and the greatest average shoot length was recorded for 10 min, while 60 min US treatment time significantly reduced growth (Figure 6(a)). MWNT-GA had greater root length (Figure 6(b)), shoot length (Figure 6(c)), and seedling dry weight (Figure 6(d)) compared to the MilliQ control and fullerol treatments. Therefore, our results indicated that the growth and biomass of tomato seedlings were enhanced by US-MWNT double exposure treatment.

## 4. Discussion

The single effects of US [23–26] and CBNM treatment alone on seed germination have been studied [4, 5, 8]. However, the effect of both of these treatments applied simultaneously to seeds has not been reported. The double exposure model was pursued to elucidate novel insights into the effect of NMs on enhancing seed coat permeability, germination, and seedling growth with the objective to develop new applications of US and NMs for the agricultural industry.

The effects of US-CBNMs double exposure were examined in this study on seed morphology (Figures 1 and 2), coat permeability (Figures 3 and 4, Table 1), seed germination (Table 2), imbibition (Figure 5), and finally seedling growth (Figure 6). Collectively, the enhanced tomato seed germination (Table 2) and seedling growth (Figure 6) were the result of combinations of US treatment duration and MWNT seed treatment, or double exposure. Short term US treatments were shown to increase the percentage germination compared to control (Table 2), and our findings are in agreement with previous studies [27–34]. Two mechanisms have been proposed for the positive biological impacts of short durations of US on seed germination. The first and most probable mechanism for US enhancement of germination is microstreaming, the mechanical or shear effect that is caused by the large and rapid oscillations in bubble size that disrupts plant cell walls, thus increasing the mass transfer and easier access of water to the interior of the cell wall structure [30, 33]. This mechanism would explain the greater water uptake during imbibition (Figure 5(a)). The second mechanism involves the creation of transient or collapse acoustic cavitations in seeds, induced by US treatment [28–31], that can cause temperature and chemical effects in tissues resulting in an enhanced enzyme activity. In support of that, US treatment increased the rate of starch hydrolysis by *α*-amylase as shown in barley seeds [32, 34]. These changes at the biochemical level would support the observed increase in germination percentage after 5 min US treatments (Table 2).

In contrast, long term (30 or 60 minutes) US treatments with either C_60_(OH)_20_ or MWNT caused significant mechanical disruption resulting in seed weight loss (Figure 5(b)) and seed coat removal (Figures 1 and 2). Though these results clearly indicate that the US treatment of seeds in the presence of NMs led to the significant disruption and removal of seed coat, penetration of NMs through the seed coat was not observed nor measured (Figures 3 and 4). Another hypothesis was that NMs or NMs with US would create channels in the seed coat, thus allowing the passage of small molecules to diffuse to the embryo. However, double exposure did not enhance seed coat permeability to the fluorescent tracer Rhod B (Figure 3 and Table 1). Our earlier report has shown the presence of a semipermeable layer in tomato seeds as an amorphous, highly compact layer located as the innermost layer of the seed coat adjacent to the endosperm [19]. Therefore, the semipermeable layer acted as a barrier for both Rhod B and NMs into seeds. In contrast, the presence of MWNT was detected in tomato seed embryos after two days of exposure to NMs when dispersed in the germinating media [5]. Therefore the uptake of NMs in the previously reported study [5] may have occurred from root uptake and not through direct penetration of the seed coat.

An increase in average seedling length and weight was only measured from the MWNT-GA treatments in comparison with the MilliQ 0 control (Figures 6(a)–6(d)). This enhanced seedling growth may be partially attributed to removal of the seed coat during the 60 min treatments, thus reducing the barrier for radicle emergence. In addition, the uptake of residual NMs on tomato seeds after treatment may have occurred after radicle emergence and thus promoted seedling growth. However, our 60 min soak method differs from the previous report [5] in which NMs that enhanced plant growth were provided in the germination media. An alternative approach that warrants further study is to apply CBNMs as a seed treatment with a seed coating technology [22].

## 5. Conclusions

Using this double exposure model, we demonstrated an increase in seed germination, root and seedling growth, and biomass of tomato plants in the presence of MWNTs. A major finding of this project was that CBNMs were not able to penetrate tomato seed coats after short 60-minute soak periods, confirmed by bright field, fluorescence, and electron microscopy examinations and micro-Raman spectroscopy. In contrast to our findings, MWNTs were stated to penetrate tomato seed coats [5] and seed coats of agronomic crops [8], and single-walled carbon nanotubes were reported to penetrate seed coats of other crop species [4]. The barrier to NM uptake by tomato seeds during imbibition was attributed to the semipermeable layer that was shown to be a barrier to movement of water-soluble compounds [19]. More recent research in our lab has shown that low molecular weight (<500), nonionic compounds with moderate lipophilic physical-chemical characteristics may diffuse into tomato seeds and thus pass through the seed coat's semipermeable layer [20, 21]. Therefore, there is a possibility that certain hydrophobic materials may be able to penetrate the seed coat and diffuse to the embryo. However, NMs may be several orders of magnitude larger than molecular weight of 500. Collectively, our results provided insight into NM movement during the early stages of tomato seed germination and highlighted seed coat permeability as a major barrier to the penetration of NMs of a particular species. The potential impact of CBNM seed treatments on both food safety and the environment is a critical subject to understand. Future research is needed to address questions on the physical modification of the seed coat by US in combination with MWNTs on seedling and plant growth and development, including yield.

## Supplementary Material

The hydrophobic nature of MWNTs required the presence of an amphiphylic-solubilizing factor for a stable suspension. GA, a type of phenolic acid, was chosen to solubilize NMs and enhance their bioavailability. The description and characterization of C60 (OH)20, MWNTs, and GA was conducted and at 50 mg/L concentration, the hydrodynamic sizes of 0.93 nm and 188.88 nm, zeta potential –51.1 mV and –29.4 mV, polydispersity index 0.52 and 0.24 were recorded for C60 (OH)20 and MWNT-GA, respectively

## Figures and Tables

**Table 1:** The effect of sonication for 0 and 60 minutes and CBNM seed treatments on the percentage of seeds with permeable seed coats as determined by diffusion of Rhodamine B to the embryo.

Treatment	Intact seeds		Punctured seeds	
	0 min	60 min	0 min	60 min
MilliQ	0.9 ± 1.2	1.7 ± 0.0	100.0 ± 0.0	100.0 ± 0.0
GA	0.0 ± 0.0	2.2 ± 1.2	100.0 ± 0.0	100.0 ± 0.0
Fullerol	0.9 ± 1.2	3.3 ± 0.0	100.0 ± 0.0	100.0 ± 0.0
MWNT-GA	1.7 ± 2.3	3.3 ± 2.3	100.0 ± 0.0	100.0 ± 0.0

Results are means ± standard errors.

**Table 2:** The effect of sonication for selected durations and CBNM seed treatments on percent germination recorded after 5 days.

Duration (min)	MilliQ	GA	Fullerol	MWNT-GA
0	93 ± 2.0	98 ± 1.6	96 ± 3.0	99 ± 1.5
5	99 ± 0.5	99 ± 0.3	99 ± 0.8	99 ± 0.8
10	96 ± 3.0	96 ± 2.2	98 ± 1.6	99 ± 0.3
30	97 ± 1.9	96 ± 1.2	98 ± 1.6	99 ± 1.5
60	96 ± 1.9	92 ± 4.5	95 ± 3.2	99 ± 1.5

Results are means ± standard error of six experiments (total 170 seeds for each treatment).
