# Supplementary material for: Tomato Seed Coat Permeability to Selected Carbon Nanomaterials and Enhancement of Germination and Seedling Growth
Source: ScientificWorldJournal. 2015 Oct 1;2015:419215. doi: 10.1155/2015/419215 (PMC4606189; doi:10.1155/2015/419215)
Supplement: Supplementary file 1 — The hydrophobic nature of MWNTs required the presence of an amphiphylic-solubilizing factor for a stable suspension. GA, a type of phenolic acid, was chosen to solubilize NMs and enhance their bioavailability. The description and characterization of C60 (OH)20, MWNTs, and GA was conducted and at 50 mg/L concentration, the hydrodynamic sizes of 0.93 nm and 188.88 nm, zeta potential –51.1 mV and –29.4 mV, polydispersity index 0.52 and 0.24 were recorded for C60 (OH)20 and MWNT-GA, respectively [file 419215.f1.pdf]

Supplemental Information.

Nanoparticle Suspension Preparations and Characterization

The hydrophobic nature of MWNTs required the presence of an amphiphylic-solubilizing factor for a stable suspension of these NMs. Gallic (GA) a type of phenolic acid, is an ideal compound to enhance the bioavailability and dispersion of NMs. Specifically, the abundance of GA in plants, its significant therapeutic properties [35, 36], and great possibility to interact with CBNMs rendered it useful in this particular study [37]. $C_{60}(OH)_{20}$ (BuckyUSA), MWNTs (Sigma, outer diameter: 40–70 nm, inner diameter: 5–40 nm, length: 0.5–2 μm, purity: 95+ %), and GA (Sigma, G7384) were used as purchased. 500 mg/L samples of all stock solutions, $C_{60}(OH)_{20}$, MWNT-GA, and GA were prepared according to the following protocols. $C_{60}(OH)_{20}$ and GA were dissolved directly into MilliQ water and sonicated for 10 min to prevent agglomeration. MWNTs were added to the GA suspensions, the concentration of each being 500 mg/mL. After probe sonication at 8W for 60 min (VC130 PB, Sonics & Materials, Inc.), the sample was vortexed, and its absorbance was measured immediately and after 24 hours (Biomate 3, λ = 400 nm). No significant difference was detected in the absorbance values, indicating that negligible sample precipitation occurred during first 24 hours (data not shown). The size distributions of the $C_{60}(OH)_{20}$, GA and MWNT-GA were determined using dynamic light scattering (Malvern, nanosizer S90; range 0.3 nm–5 μm, resolution 1 nm) at room temperature (see SI, Figure S1). The major peak values at ∼1.2 nm and 227 nm were identified for the 50 mg/L of $C_{60}(OH)_{20}$, MWNT-GA suspensions accordingly. Good dispersability and stability of $C_{60}(OH)_{20}$ and MWNT-GA (see for example, Figure S1) suspensions were confirmed by their negative zeta potentials, of -51.1 mV and -29.4 mV, respectively (ZetaSizerNano ZS) in MilliQ water (see SI, Table S1). The solubility of NMs is attributed to the formation of the hydrogen bonds between the hydroxyl groups of the $C_{60}(OH)_{20}$ and oxygen atoms of water [38, 39], and hydrophobic interaction and pi-pi stacking between the hydrophobic moieties of GA and the surface of MWNT (Salonen *et al.*, 2008). The key physical parameters of all suspensions are summarized in the supporting information (see SI, Table S1). MWNT-GA, $C_{60}(OH)_{20}$ and GA suspensions were diluted into nominal concentrations 50 mg/L using MilliQ and used in all experiments.

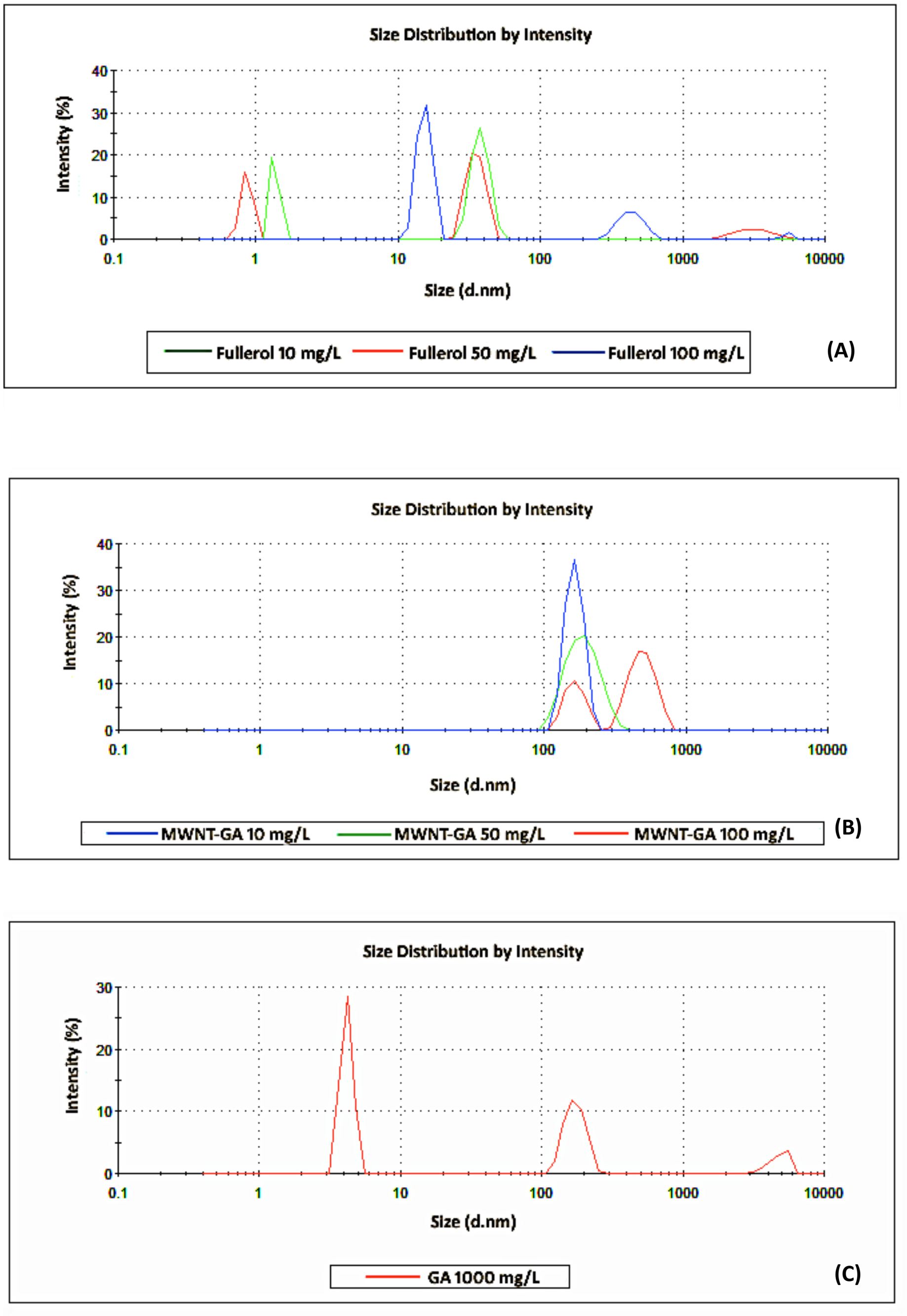

Figure S1. $C_{60}(OH)_{20}$, MWNT-GA and GA size distribution measured by a nanosizer (Zetasizer S90).

The parameter "size" as determined by the Zetasizer refers to the hydrodynamic diameter of a Rayleigh particle. According to Rayleigh's approximation, the intensity of scattering of a particle is proportional to the sixth power of its diameter.

(A) The sample of $C_{60}(OH)_{20}$ in Milli-Q water displayed size peaks at 1.36 nm (10 mg/L), 0.93 nm (50 mg/L) and 10.77 nm (100 mg/L). The registered larger particles sizes were possibly due to the nanoparticle aggregation in Milli-Q water through the formation of hydrogen bonds in Milli-Q water or possibly due possible contamination in the suspension. Major size distribution peak of 50 mg/L $C_{60}(OH)_{20}$ was 1.2 ± 0.4 nm (PdI = 0.516 ± 0.090)

(B) The sample of MWNT-GA in Milli-Q water displayed size distribution peaks at 172.06 nm (10 mg/L), 188.88 nm (50 mg/L) and 180.53 nm and 430.33 nm (100 mg/L). Major size distribution peak of 50 mg/L MWNT-GA was 227.1 ± 38.7 nm (PdI = 0.237±0.069).

(C) The size distribution of GA of 10, 50, 100 mg/L concentrations were not detectable (count rate was too low). The sample of GA 1000mg/L displayed the major distribution peak at 5.59 ± 1.10 nm (PdI = 0.376 ± 0.042). The registered larger particles sizes were possibly due to the GA aggregation in Milli-Q water or presence of dust.

All measurements were done in triplicate.

Table S1. Characterization of GA suspension and $C_{60}(OH)_{20}$, MWNT-GA nanomaterials.

	**Hydrodynamic diameter (nm)**	**Zeta Potential (mV)**	**Polydispersity Index**
**GA**	5.61 ± 1.12	-2.33 ± 1.37	0.37 ± 0.04
**Fullerol**	1.17 ± 0.36	-51.05 ± 4.85	0.52 ± 0.09
**MWNT-GA**	223.08 ± 38.72	-29.40 ± 1.54	0.24 ± 0.07

Results represent mean ± standard deviation of three experiements.
